# Bis(2-amino­pyridinium) 2,5-dicarb­oxy­benzene-1,4-dicarboxyl­ate

**DOI:** 10.1107/S1600536812017254

**Published:** 2012-04-25

**Authors:** V. H. Rodrigues, Mohammad Hakimi, Elham Motieiyan

**Affiliations:** a CEMDRX, Department of Physics, University of Coimbra, P-3004-516 Coimbra, Portugal; b Department of Chemistry, Payame Noor University, 19395-4697 Tehran, Iran

## Abstract

In the title compound, 2C_5_H_7_N_2_^+^·C_10_H_4_O_8_^2−^, the 2-amino­pyridinium (2-apyH) cation and 2,5-dicarb­oxy­benzene-1,4-dicarboxyl­ate (btcH2) anion are both nearly planar, with r.m.s. deviations of 0.015 and 0.050 Å, respectively. The angle between the latter least-squares planes is 17.68 (9)°. The overall crystal structure results from the packing of two-dimensional networks, formed by alternating 2-apyH and btcH2 linked by hydrogen bonds, parallel to (100).

## Related literature

For similar and most common conformations of 2-amino­pyridinium, see: Guelmami & Jouini (2011 ▶); Chitra *et al.* (2008 ▶); Quah *et al.* (2008 ▶); Bis & Zaworotko (2005 ▶); Büyükgüngör & Odabas˛ogˇlu (2002 ▶); Odabas˛ogˇlu *et al.* (2003 ▶); Acheson (1967 ▶). For similar and most common conformations of 2,5-dicarb­oxy­benzene-1,4-dicarboxyl­ate, see: Dong *et al.* (2011 ▶); Wang & Tang (2010 ▶). For graph-set analysis of hydrogen-bond patterns in organic crystals, see: Etter *et al.* (1990 ▶). 
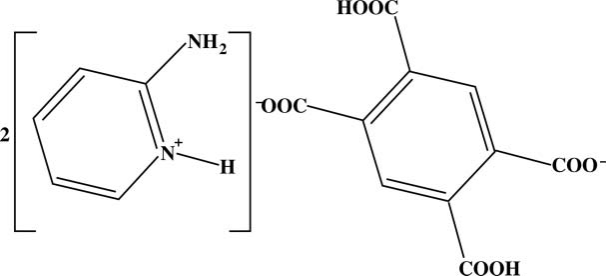


## Experimental

### 

#### Crystal data

2C_5_H_7_N_2_^+^·C_10_H_4_O_8_^2−^
*M* *_r_* = 442.38
Monoclinic, 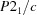

*a* = 4.0165 (1) Å
*b* = 10.8098 (4) Å
*c* = 21.4036 (7) Å
β = 99.535 (2)°
*V* = 916.45 (5) Å^3^
*Z* = 2
Mo *K*α radiation
μ = 0.13 mm^−1^
*T* = 273 K
0.3 × 0.2 × 0.15 mm

#### Data collection

Bruker–Nonius APEXII CCD area-detector diffractometer
Absorption correction: multi-scan (*SADABS*; Sheldrick, 2003 ▶) *T*_min_ = 0.755, *T*_max_ = 1.000
18838 measured reflections
2209 independent reflections
1652 reflections with *I* > 2σ(*I*)
*R*_int_ = 0.024

#### Refinement

*R*[*F* ^2^ > 2σ(*F* ^2^)] = 0.037
*wR*(*F* ^2^) = 0.102
*S* = 1.01
2209 reflections
158 parameters
H atoms treated by a mixture of independent and constrained refinement
Δρ_max_ = 0.20 e Å^−3^
Δρ_min_ = −0.15 e Å^−3^

### 

Data collection: *APEX2* (Bruker–Nonius, 2004 ▶); cell refinement: *SAINT* (Bruker, 2003 ▶); data reduction: *SAINT*; program(s) used to solve structure: *SHELXS97* (Sheldrick, 2008 ▶); program(s) used to refine structure: *SHELXL97* (Sheldrick, 2008 ▶); molecular graphics: *PLATON* (Spek, 2009 ▶); software used to prepare material for publication: *SHELXL97*.

## Supplementary Material

Crystal structure: contains datablock(s) I, global. DOI: 10.1107/S1600536812017254/bt5880sup1.cif

Structure factors: contains datablock(s) I. DOI: 10.1107/S1600536812017254/bt5880Isup2.hkl

Supplementary material file. DOI: 10.1107/S1600536812017254/bt5880Isup3.cml

Additional supplementary materials:  crystallographic information; 3D view; checkCIF report

## Figures and Tables

**Table 1:** Hydrogen-bond geometry (Å, °)

*D*—H⋯*A*	*D*—H	H⋯*A*	*D*⋯*A*	*D*—H⋯*A*
N1—H1*A*⋯O2	0.913 (16)	1.904 (17)	2.7852 (15)	161.6 (15)
N1—H1*A*⋯O1	0.913 (16)	2.478 (16)	3.2413 (16)	141.4 (13)
N2—H2*A*⋯O3^i^	0.831 (19)	2.125 (19)	2.9520 (17)	173.0 (17)
N2—H2*B*⋯O1	0.880 (19)	2.14 (2)	2.9759 (17)	157.6 (16)
O4—H4*A*⋯O1	1.06 (2)	1.31 (2)	2.3766 (15)	176.8 (17)

Symmetry code: (i) 
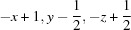
.

**Fig. 1.**
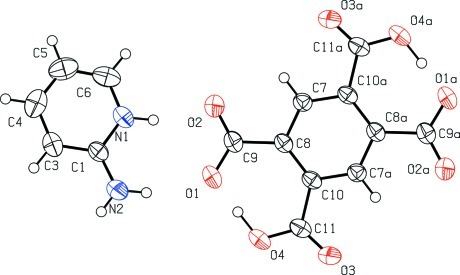
ORTEPII plot of the title compound. Displacement ellipsoids are drawn at the 50% level.

**Fig. 2.**
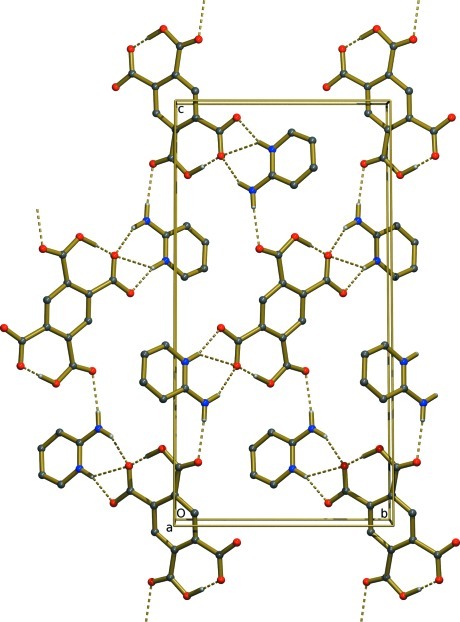
Representation of the two-dimensional networks, paralell to the (100) planes, of H-bonded molecules.

## supplementary crystallographic information

### Comment

This work is a further contribution to the broad family of structural studies of 2-aminopyridinium (2-apyH) systems with hydrogen-bond donors. A considerable number of analogous materials formed from 2-aminopyridine and a given carboxylic acid has already been reported (Guelmami & Jouini, 2011; Chitra *et al.*, 2008; Quah *et al.*, 2008; Bis & Zaworotko, 2005; Büyükgüngör & Odabas&ogon;og&caron;lu, 2002; Odabas&ogon;og&caron;lu *et al.*, 2003; *etc*). This is due to the fact that 2-aminopyridine is protonated in acidic solutions. It is well known that the bonding of the H atom to the ring N atom of 2-aminopyridine, and not to the amino N atom, produces an ion for which an additional resonance structure must be considered (Acheson, 1967). We have inferred the positive charge in the 2-apyH ion lies on the amino group based on a difference fourier map, a common practice when allowed by the quality of the collected intensities. The charge state, related to the hydrogen loss, in each of the two candidate carboxylic acid groups belonging to the assymetric unit was also inferred from a difference map and further reinforced by analysis of the C–O bond lengths.

Ellucidation of the numbering scheme and a view of the H-bonds giving rise to two-dimensional networks parallel to (100) are shown in Figs. 1 and 2, respectively. Both the 2-apyH cation and 2,5-dicarboxybenzene-1,4-dicarboxylate (btcH2) anion are nearly planar, with r.m.s. deviations of 0.015 and 0.050 A, respectively. The angle between the latter idealized planes is 17.68 (9)°. The two-dimensional networks are formed by alternating 2-apyH and btcH2 linked by H-bonds and include all H-bonds found. The first order network describing the H-bonding in the title compound is N_1_=4DS(7), as established by applying the rules of graph-set analysis of hydrogen-bond patterns in organic crystals (Etter *et al.*, 1990).

Similar and most common conformations of 2,5-dicarboxybenzene-1,4-dicarboxylate were described by Dong *et al.* (2011) and Wang & Tang (2010).

### Experimental

A solution of 0.254 g (1 mmol) benzene-1,2,4,5-tetracarboxylicacid in methanol (10 ml) was added to a solution of 2-aminopyridine (0.1 g, 1 mmol) in water (15 ml), and refluxed for 1 h. The resulting solution was light yellow in colour. After slow evaporation of the solvent at room-temperature colorless prisms of the compound were obtained.

### Refinement

The structure was solved by direct methods using *SHELXS97* (Sheldrick, 2008). H atoms bound to aromatic C were placed at idealized positions and refined as riding, with C—H=0.93 (Sheldrick, 2008); amine and carboxyl H atoms were found from a difference fourier map and their coordinates refined freely. *U*_iso_(H) was fixed to 1.2 times *U*_eq_ of the heavy atom they are bonded to, for all hydrogen atoms.

Examination of the crystal structure with *PLATON* (Spek, 2009) showed that there are no solvent-accessible voids in the crystal lattice.

### Crystal data

**Table:**

2C_5_H_7_N_2_^+^·C_10_H_4_O_8_^2^^−^	*F*(000) = 460
*M**_r_* = 442.38	*D*_x_ = 1.603 Mg m^−^^3^
Monoclinic, *P*2_1_/*c*	Mo *K*α radiation, λ = 0.71073 Å
Hall symbol: -P 2ybc	Cell parameters from 7209 reflections
*a* = 4.0165 (1) Å	θ = 2.7–24.9°
*b* = 10.8098 (4) Å	µ = 0.13 mm^−^^1^
*c* = 21.4036 (7) Å	*T* = 273 K
β = 99.535 (2)°	Block, yellow
*V* = 916.45 (5) Å^3^	0.3 × 0.2 × 0.15 mm
*Z* = 2	

### Data collection

**Table:**

Bruker–Nonius APEXII CCD area-detector diffractometer	2209 independent reflections
Radiation source: fine-focus sealed tube	1652 reflections with *I* > 2σ(*I*)
Graphite monochromator	*R*_int_ = 0.024
φ and ω scans	θ_max_ = 28.2°, θ_min_ = 1.9°
Absorption correction: multi-scan (*SADABS*; Sheldrick, 2003)	*h* = −4→5
*T*_min_ = 0.755, *T*_max_ = 1.000	*k* = −13→14
18838 measured reflections	*l* = −27→23

### Refinement

**Table:**

Refinement on *F*^2^	Secondary atom site location: difference Fourier map
Least-squares matrix: full	Hydrogen site location: inferred from neighbouring sites
*R*[*F*^2^ > 2σ(*F*^2^)] = 0.037	H atoms treated by a mixture of independent and constrained refinement
*wR*(*F*^2^) = 0.102	*w* = 1/[σ^2^(*F*_o_^2^) + (0.0519*P*)^2^ + 0.1584*P*] where *P* = (*F*_o_^2^ + 2*F*_c_^2^)/3
*S* = 1.01	(Δ/σ)_max_ < 0.001
2209 reflections	Δρ_max_ = 0.20 e Å^−^^3^
158 parameters	Δρ_min_ = −0.15 e Å^−^^3^
0 restraints	Extinction correction: *SHELXL97* (Sheldrick, 2008), Fc^*^=kFc[1+0.001xFc^2^λ^3^/sin(2θ)]^-1/4^
Primary atom site location: structure-invariant direct methods	Extinction coefficient: 0.014 (3)

### Special details

**Table:**

Geometry. All e.s.d.'s (except the e.s.d. in the dihedral angle between two l.s. planes) are estimated using the full covariance matrix. The cell e.s.d.'s are taken into account individually in the estimation of e.s.d.'s in distances, angles and torsion angles; correlations between e.s.d.'s in cell parameters are only used when they are defined by crystal symmetry. An approximate (isotropic) treatment of cell e.s.d.'s is used for estimating e.s.d.'s involving l.s. planes.
Refinement. Refinement of *F*^2^ against ALL reflections. The weighted *R*-factor *wR* and goodness of fit *S* are based on *F*^2^, conventional *R*-factors *R* are based on *F*, with *F* set to zero for negative *F*^2^. The threshold expression of *F*^2^ > σ(*F*^2^) is used only for calculating *R*-factors(gt) *etc*. and is not relevant to the choice of reflections for refinement. *R*-factors based on *F*^2^ are statistically about twice as large as those based on *F*, and *R*- factors based on ALL data will be even larger.

### Fractional atomic coordinates and isotropic or equivalent isotropic displacement parameters (Å^2^)

**Table:**

	*x*	*y*	*z*	*U*_iso_*/*U*_eq_	
N1	1.1547 (3)	0.04940 (11)	0.38178 (5)	0.0424 (3)	
H1A	1.040 (4)	0.1095 (15)	0.3993 (8)	0.051*	
C1	1.1679 (3)	0.04905 (12)	0.32019 (6)	0.0370 (3)	
N2	1.0229 (4)	0.14007 (12)	0.28496 (6)	0.0511 (3)	
H2A	1.021 (4)	0.1363 (16)	0.2461 (9)	0.061*	
H2B	0.916 (5)	0.1990 (16)	0.3019 (9)	0.061*	
C3	1.3390 (4)	−0.04878 (13)	0.29700 (7)	0.0469 (4)	
H3	1.3508	−0.0532	0.2540	0.056*	
C4	1.4882 (4)	−0.13741 (15)	0.33704 (9)	0.0569 (4)	
H4	1.6042	−0.2019	0.3215	0.068*	
C5	1.4684 (4)	−0.13228 (16)	0.40058 (9)	0.0587 (4)	
H5	1.5712	−0.1922	0.4285	0.070*	
C6	1.2984 (4)	−0.03922 (15)	0.42096 (7)	0.0523 (4)	
H6	1.2786	−0.0356	0.4636	0.063*	
C7	0.7030 (3)	0.40755 (11)	0.52514 (6)	0.0325 (3)	
H7	0.8444	0.3440	0.5427	0.039*	
C8	0.5869 (3)	0.40386 (11)	0.46123 (5)	0.0312 (3)	
C9	0.7148 (3)	0.29317 (12)	0.42838 (6)	0.0380 (3)	
O1	0.6529 (3)	0.28618 (10)	0.36938 (5)	0.0588 (3)	
O2	0.8806 (3)	0.21344 (9)	0.45984 (5)	0.0591 (3)	
C10	0.3760 (3)	0.50071 (11)	0.43487 (5)	0.0315 (3)	
C11	0.2130 (3)	0.52081 (12)	0.36717 (6)	0.0386 (3)	
O3	0.0283 (3)	0.60888 (10)	0.35332 (5)	0.0571 (3)	
O4	0.2721 (3)	0.44339 (10)	0.32502 (4)	0.0556 (3)	
H4A	0.448 (5)	0.3738 (17)	0.3439 (8)	0.067*	

### Atomic displacement parameters (Å^2^)

**Table:**

	*U*^11^	*U*^22^	*U*^33^	*U*^12^	*U*^13^	*U*^23^
N1	0.0464 (7)	0.0473 (7)	0.0338 (6)	−0.0050 (5)	0.0072 (5)	−0.0078 (5)
C1	0.0391 (7)	0.0395 (7)	0.0316 (7)	−0.0064 (5)	0.0038 (5)	−0.0049 (5)
N2	0.0690 (9)	0.0481 (7)	0.0339 (6)	0.0058 (6)	0.0017 (6)	−0.0040 (6)
C3	0.0463 (8)	0.0510 (9)	0.0452 (8)	−0.0037 (7)	0.0129 (6)	−0.0118 (7)
C4	0.0450 (8)	0.0447 (8)	0.0820 (12)	0.0006 (7)	0.0131 (8)	−0.0058 (8)
C5	0.0511 (9)	0.0552 (9)	0.0664 (11)	−0.0078 (7)	0.0001 (8)	0.0187 (8)
C6	0.0516 (9)	0.0648 (10)	0.0387 (8)	−0.0144 (8)	0.0025 (7)	0.0090 (7)
C7	0.0367 (6)	0.0310 (6)	0.0303 (6)	−0.0027 (5)	0.0067 (5)	0.0019 (5)
C8	0.0349 (6)	0.0316 (6)	0.0284 (6)	−0.0069 (5)	0.0091 (5)	−0.0015 (5)
C9	0.0437 (7)	0.0364 (7)	0.0351 (7)	−0.0050 (6)	0.0102 (6)	−0.0056 (5)
O1	0.0832 (8)	0.0600 (7)	0.0336 (6)	0.0173 (6)	0.0113 (5)	−0.0114 (5)
O2	0.0841 (8)	0.0469 (6)	0.0453 (6)	0.0203 (6)	0.0074 (5)	−0.0048 (5)
C10	0.0358 (6)	0.0336 (6)	0.0259 (6)	−0.0087 (5)	0.0069 (5)	0.0005 (5)
C11	0.0451 (7)	0.0429 (7)	0.0275 (6)	−0.0065 (6)	0.0055 (5)	0.0006 (5)
O3	0.0777 (7)	0.0550 (7)	0.0336 (6)	0.0151 (6)	−0.0053 (5)	0.0017 (4)
O4	0.0763 (8)	0.0634 (7)	0.0257 (5)	0.0096 (6)	0.0045 (5)	−0.0065 (5)

### Geometric parameters (Å, º)

**Table:**

N1—C1	1.3281 (17)	C6—H6	0.9300
N1—C6	1.3392 (19)	C7—C8	1.3707 (17)
N1—H1A	0.913 (16)	C7—C10^i^	1.3809 (17)
C1—N2	1.3154 (19)	C7—H7	0.9300
C1—C3	1.3964 (19)	C8—C10	1.4049 (18)
N2—H2A	0.831 (19)	C8—C9	1.5197 (17)
N2—H2B	0.880 (19)	C9—O2	1.2212 (16)
C3—C4	1.357 (2)	C9—O1	1.2483 (16)
C3—H3	0.9300	C10—C7^i^	1.3809 (17)
C4—C5	1.377 (2)	C10—C11	1.5037 (17)
C4—H4	0.9300	C11—O3	1.2133 (17)
C5—C6	1.329 (2)	C11—O4	1.2811 (16)
C5—H5	0.9300	O4—H4A	1.06 (2)
			
C1—N1—C6	122.39 (13)	C5—C6—H6	119.1
C1—N1—H1A	121.0 (10)	N1—C6—H6	119.1
C6—N1—H1A	116.6 (10)	C8—C7—C10^i^	124.36 (12)
N2—C1—N1	118.70 (13)	C8—C7—H7	117.8
N2—C1—C3	124.06 (13)	C10^i^—C7—H7	117.8
N1—C1—C3	117.24 (13)	C7—C8—C10	117.52 (11)
C1—N2—H2A	117.8 (12)	C7—C8—C9	113.54 (11)
C1—N2—H2B	120.4 (12)	C10—C8—C9	128.93 (11)
H2A—N2—H2B	121.5 (17)	O2—C9—O1	120.93 (12)
C4—C3—C1	120.15 (14)	O2—C9—C8	119.73 (11)
C4—C3—H3	119.9	O1—C9—C8	119.33 (12)
C1—C3—H3	119.9	C7^i^—C10—C8	118.12 (11)
C3—C4—C5	120.25 (15)	C7^i^—C10—C11	112.67 (11)
C3—C4—H4	119.9	C8—C10—C11	129.20 (11)
C5—C4—H4	119.9	O3—C11—O4	121.18 (12)
C6—C5—C4	118.13 (15)	O3—C11—C10	119.99 (12)
C6—C5—H5	120.9	O4—C11—C10	118.83 (12)
C4—C5—H5	120.9	C11—O4—H4A	112.5 (9)
C5—C6—N1	121.83 (15)		
			
C6—N1—C1—N2	−179.42 (13)	C10—C8—C9—O2	−175.39 (13)
C6—N1—C1—C3	0.14 (19)	C7—C8—C9—O1	−173.10 (12)
N2—C1—C3—C4	178.46 (14)	C10—C8—C9—O1	5.7 (2)
N1—C1—C3—C4	−1.1 (2)	C7—C8—C10—C7^i^	−0.56 (18)
C1—C3—C4—C5	0.8 (2)	C9—C8—C10—C7^i^	−179.36 (11)
C3—C4—C5—C6	0.5 (2)	C7—C8—C10—C11	−179.96 (11)
C4—C5—C6—N1	−1.4 (2)	C9—C8—C10—C11	1.2 (2)
C1—N1—C6—C5	1.1 (2)	C7^i^—C10—C11—O3	−1.71 (17)
C10^i^—C7—C8—C10	0.60 (19)	C8—C10—C11—O3	177.72 (13)
C10^i^—C7—C8—C9	179.58 (11)	C7^i^—C10—C11—O4	178.55 (12)
C7—C8—C9—O2	5.76 (17)	C8—C10—C11—O4	−2.01 (19)

Symmetry code: (i) −*x*+1, −*y*+1, −*z*+1.

### Hydrogen-bond geometry (Å, º)

**Table:**

*D*—H···*A*	*D*—H	H···*A*	*D*···*A*	*D*—H···*A*
N1—H1*A*···O2	0.913 (16)	1.904 (17)	2.7852 (15)	161.6 (15)
N1—H1*A*···O1	0.913 (16)	2.478 (16)	3.2413 (16)	141.4 (13)
N2—H2*A*···O3^ii^	0.831 (19)	2.125 (19)	2.9520 (17)	173.0 (17)
N2—H2*B*···O1	0.880 (19)	2.14 (2)	2.9759 (17)	157.6 (16)
O4—H4*A*···O1	1.06 (2)	1.31 (2)	2.3766 (15)	176.8 (17)

Symmetry code: (ii) −*x*+1, *y*−1/2, −*z*+1/2.
